# *MAML2*-Rearranged Primary Central Mucoepidermoid Carcinoma of the Mandible as an Incidental Finding: A Case Report and Review of the Literature of Molecularly Confirmed Cases

**DOI:** 10.1155/2023/7764292

**Published:** 2023-04-11

**Authors:** Sarah E. Aguirre, Donald Tyler, Adepitan A. Owosho

**Affiliations:** ^1^Department of Diagnostic Sciences, College of Dentistry, The University of Tennessee Health Sciences Center, Memphis, TN, USA; ^2^Joint Base San Antonio-Lackland Air Force Base, San Antonio, TX, USA; ^3^Department of Otolaryngology—Head & Neck Surgery, College of Medicine, The University of Tennessee Health Sciences Center, Memphis, TN, USA

## Abstract

This report presents an extremely rare case of *MAML2*-rearranged primary central mucoepidermoid carcinoma (MEC) of the mandible that was discovered as an incidental finding. Our review of the literature identified 36 cases of *MAML2-*rearranged intraosseous lesions of the jaw (30 central MECs, 5 odontogenic cysts with mucous prosoplasia, and 1 glandular odontogenic cyst). Given the therapeutic indications for a diagnosis of MEC (a malignant neoplasm), *MAML2* rearrangement should be confirmed in suspected cases of central MEC.

## 1. Introduction

Mucoepidermoid carcinoma (MEC) is the most common malignant primary salivary gland tumor and accounts for 5–10% of all salivary gland tumors [1, 2]. MEC typically arises from the major or minor salivary glands. Central MEC (CMEC) also known as intraosseous MEC is rare, comprising only 2–3% of all MECs reported [1, 3, 4], with over 200 cases of CMEC reported in the literature. While the salivary gland MEC was first described in 1895 by Volkmann [5], its central counterpart was not reported until 1939 by Lepp in a 66-year-old female patient (according to Pires et al.) [1, 6]. *MAML2* rearrangement with fusion partners *CRTC1*(*MECT1*) and rarely *CRTC3* (*MECT3*) has been found in over 75% of salivary gland MECs [7]. CRTC1/3-MAML2 fusions are specific to MEC and absent in other tumors of salivary gland origin [8, 9]. Few reports have documented the presence of *MAML2* rearrangement in CMEC [10–18].

CMEC occurs more commonly in the mandible with the premolar–molar–angle region as the most common site of occurrence, and the majority of CMECs occur in the fifth to seventh decade of life with a predilection for females [1, 2, 19]. Common symptoms associated with CMEC are swelling and pain, with less common presentations including paraesthesia, tooth mobility, and trismus [1, 19, 20]. Radiographically, CMEC presents as a unilocular or multilocular radiolucency, exhibiting considerable radiographic overlap with odontogenic cysts and tumors [1, 3, 20, 21]. However, glandular odontogenic cyst (GOC), a common radiographic and histomorphologic mimicker, has been shown to lack *MAML2* rearrangement, discrediting the etiologic relationship between GOC and CMEC [12]. We report a rare case of primary CMEC of the mandible with *MAML2* rearrangement in a 75-year-old female, adding to the few reports of documented *MAML2* rearrangement in CMEC.

## 2. Case Report

**Figure 1:**
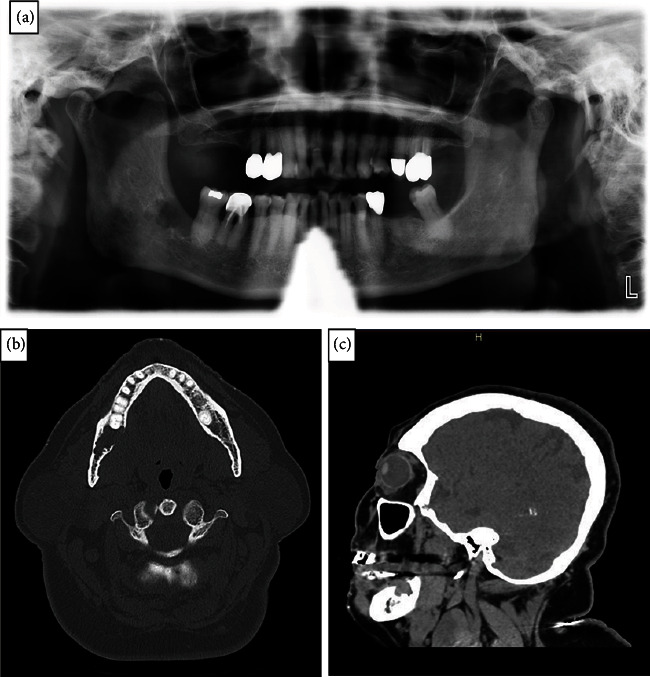
Radiologic images of a *MAML2-*rearranged central mucoepidermoid carcinoma. (a) Panoramic radiograph showing an ill-defined unilocular radiolucency distal to tooth #31. (b) Axial view of a CT-scan shows a unilocular radiolucency distal to tooth #31 with erosion of the lingual cortical plate and thinning of the buccal cortical plate. (c) Sagittal view of a CT-scan shows a unilocular radiolucency.

A 75-year-old female patient was initially admitted to the Brooke Army Medical Center (BAMC) emergency room, San Antonio, for a motor vehicle accident when a neck lymphadenopathy was identified. Because of the lymphadenopathy and evaluation/extraction of non-restorable teeth #14 and #30, she was referred to the Oral & Maxillofacial Surgery Unit of BAMC from the general surgery unit of BAMC and a private dental office, respectively. The patient stated that the right neck swelling had progressively increased in size, prompting her to seek care with the general surgery unit. Extraoral examination was negative for facial asymmetry, erythema, and tenderness on palpation. An appreciable enlarged cervical lymph node of the neck was fixed and non-tender on palpation. Panoramic radiographic imaging revealed an asymptomatic ill-defined radiolucent lesion distal to tooth #31, without association with the apices, and no evidence of root resorption (Figure 1(a)). Intraorally, tooth #31 was not mobile, and the overlying tissue distal to tooth #31 was intact, normal in color, and without signs of infection, or purulence, but it was tender to palpation. A CT-scan of the skull and jaws showed the mandibular radiolucency measuring 23 mm in widest diameter, erosion of the lingual cortical plate, thinning of the buccal cortical plate, and absence of bucco-lingual bony expansion (Figures 1(b) and 1(c)).

**Figure 2:**
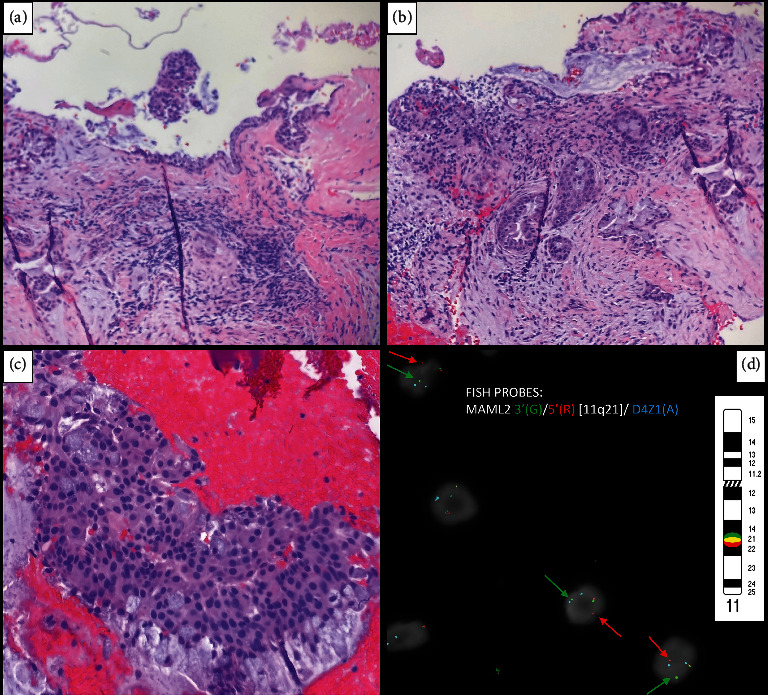
Histopathologic photomicrographs of a *MAML2*-rearranged central mucoepidermoid carcinoma. (a) Epithelial lined cyst wall with infiltrating nests of admixed epidermoid and mucous cells (H&E ×40). (b) Infiltrating nests of epidermoid and admixed mucous cells (H&E ×100). (c) Infiltrating nest of admixed epidermoid and mucous cells (H&E ×400). (d) Split signals indicating positive *MAML2* rearrangement by fluorescence in-situ hybridization [Mayo Clinic Laboratories, Laboratory Developed Test 11q21(3′*MAML2*,5′*MAML2*) break-apart probe].

An incisional biopsy of the lesion was performed, and histopathologic examination revealed an epithelial-lined cyst wall with infiltrating nest of an admixture of epidermoid and mucous cells with a predominant mucinous component (Figures 2(a), 2(b), and 2(c)). The following differential diagnoses were considered: CMEC, GOC, and primary intraosseous mucinous adenocarcinoma. To arrive at a definitive diagnosis, tissue was sent to the Mayo Clinic Genomics Laboratory in Rochester, MN, USA, for molecular testing. The *MAML2* rearrangement was confirmed by fluorescence in-situ hybridization (Figure 2(d)), with 54% of examined cells translocated. A diagnosis of low-grade CMEC was rendered.

**Figure 3:**
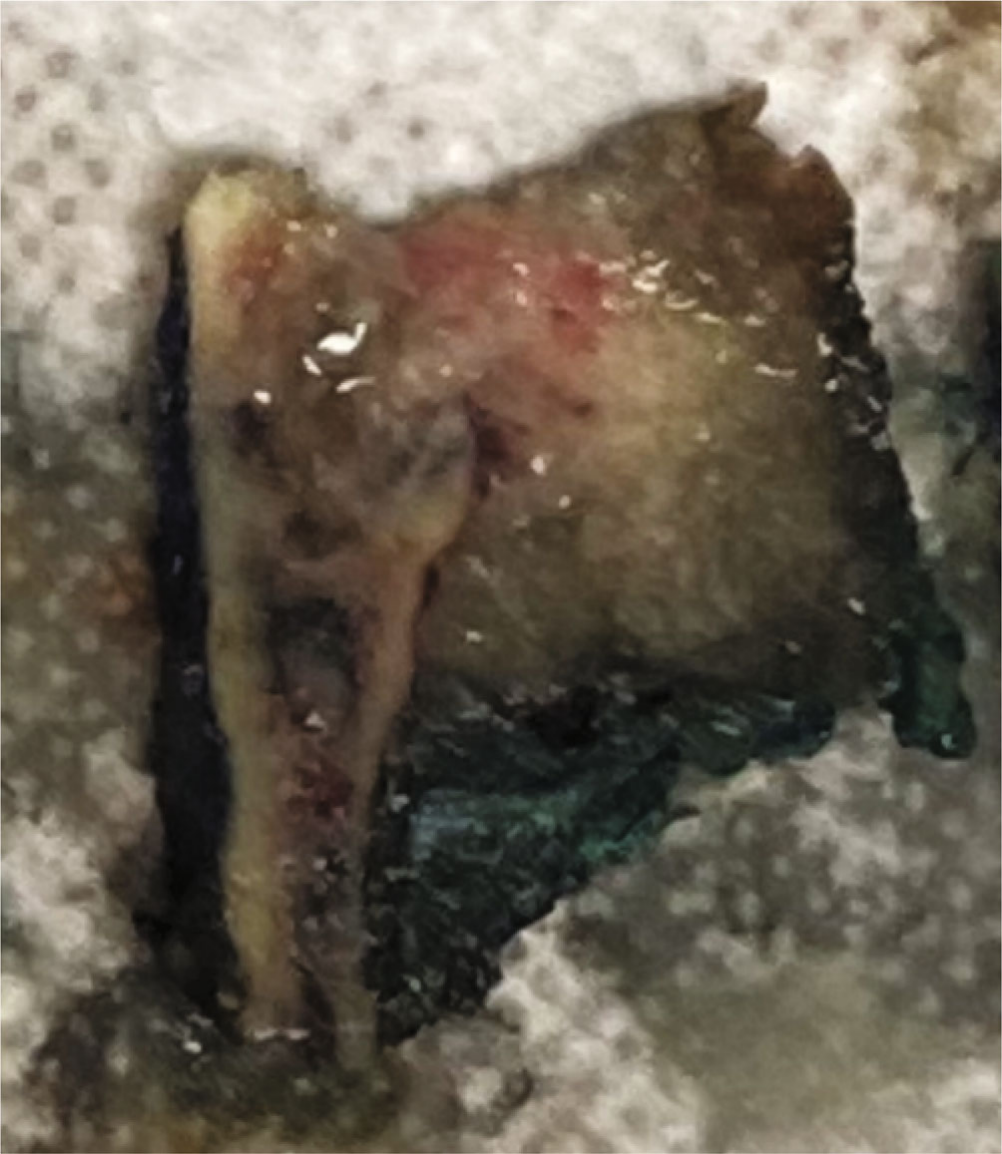
Gross evaluation of cut sections of a *MAML2*-rearranged central mucoepidermoid carcinoma of the mandible revealed a cystic cavity filled with gelatinous material.

The patient underwent radical resection, including right partial mandibulectomy, partial resection of the adjacent floor of the mouth, and right selective neck dissection of levels 2 through 4. Gross evaluation of cut sections of the mandible revealed a cystic cavity filled with gelatinous material (Figure 3). Final histopathologic examination was consistent with low-grade CMEC with soft tissue extension, perineural invasion, benign submandibular gland with chronic sialadenitis, and 2 of the 19 lymph nodes evaluated in right neck level 2 were positive for MEC. The patient's tumor was staged T3: N3b. The diagnosis of low-grade MEC was made based on the overwhelming cystic component, absence of necrosis, no to very low mitotic count per 10 high power field, and anaplasia absent.

## 3. Discussion

Primary CMEC is a rare salivary gland malignancy occurring in the jaws. The pathogenesis of CMEC within jaw bones is not fully understood, and several theories have been postulated [1, 3, 22–24]. First, neoplastic transformation of entrapped mucous glands during the development of the jaw [24]. Second, neoplastic transformation of developmentally displaced ectopic salivary gland tissue in the jaw [23]. Third, neoplastic transformation of mucous cells in the pluripotent epithelial lining of pre-existing benign odontogenic cysts [1, 3, 22]. This latter theory is supported by the fact that Eversole et al. found approximately 50% of mandibular CMEC is associated with a dental cyst and/or impacted teeth, and Brookstone and Huvos reported similar findings in 32% of cases [1, 3]. Our case was not associated with a dental cyst or an impacted tooth. The main symptoms associated with CMEC are swelling, pain, and paraesthesia [19, 20]. The patient reported none of these symptoms in this case, as this was an incidental finding. Lack of associated symptoms may be credited to early detection, as the patient was under routine dental care.

CRTC1/3-MAML2 fusions are specific to MEC as a tumor of salivary gland origin, with over 75% of salivary gland MECs harboring the fusion transcripts [7]. *MAML2* rearrangement in MEC is not specific to salivary gland origin, as MEC of other origins, such as thymus, lung, lacrimal gland, and uterine cervix, have also demonstrated *MAML2* rearrangement [25–28]. Our literature review only identified nine reports/studies documenting the presence of *MAML2* rearrangement in CMEC in 30 cases (Table 1) [10–18]. Of these, site and demographic information are provided for 25 cases [10, 11, 13–18]. The mandible represents 64% of cases, with 36% occurring in the maxilla [10, 11, 13–18]. There is a broad age range, from 20 to 85 years, which seems to separate into two peaks; 48% of cases are in patients under 32 years of age (average of 27.5 years), and 44% of cases are in individuals over 57 years of age (average of 68.2 years) [10, 11, 13–18]. Twenty cases report the histopathologic grade, with 55% characterized as low-grade and 45% characterized as intermediate-grade [10, 11, 14–18]. Two publications reporting on multiple CMECs demonstrated the *MAML2* rearrangement in 100% of their tested cases [12, 13], whereas Bell et al. and Wang et al. reported the rearrangement in 50% and 70% of tested CMECs, respectively [14, 18]. In the study by Argyris et al., five out of nine odontogenic cysts with mucous prosoplasia also demonstrated *MAML2* rearrangement [13]. With our case, the total number of molecularly confirmed cases of CMEC comes to 31 out of the over 200 cases reported in the literature. Greer et al. [29] reported *MAML2* rearrangement in a GOC case that demonstrated 7 of the 10 histologic parameters used to diagnose a GOC by Fowler et al. [30]. It is well documented that histologic and radiographic features are not conclusive enough to make a distinction between CMEC and odontogenic cyst with mucous prosoplasia/GOC [12, 13, 16, 22, 31, 32]. Recent studies have shown that MEC of different histologic variants (devoid of epidermoid cells, ciliated, Warthin-like, clear/oncocytic, sclerosing, mucinous, and spindle) have demonstrated *MAML2* rearrangement [33–35]. These histologic variations may be present in CMEC, and these *MAML2*-rearranged reported cases of odontogenic cysts with mucous prosoplasia and GOC may represent variations of CMEC.

In summary, we report an extremely rare case of *MAML2*-rearranged primary intraosseous MEC of the mandible that was discovered as an incidental radiographic finding. Our review of the literature identified 36 cases of *MAML2-*rearranged intraosseous lesions of the jaw (30 CMECs, 5 odontogenic cysts with mucous prosoplasia, and 1 GOC). Given the therapeutic indication for a diagnosis of MEC (a malignant neoplasm), *MAML2* rearrangement should be confirmed in suspected cases of CMEC. However, a negative *MAML2* rearrangement should not rule out a CMEC.

## Figures and Tables

**Table 1:** *MAML2-*rearranged central mucoepidermoid carcinoma reported in the literature.

References	Age (years)	Gender	Location	Grade
Khan et al. [10]	20	M	Mandible	NP
Bell et al. [11]	30	M	Mandibular ramus	Intermediate
Bishop et al. [12]	NP	NP	NP	NP
	NP	NP	NP	NP
	NP	NP	NP	NP
	NP	NP	NP	NP
	NP	NP	NP	NP
Argyris et al. [13]	20	F	Mandible	NP
	30	F	Mandible	NP
	85	F	Mandible	NP
Bell et al. [14]	31	M	Maxilla	Intermediate
	71	F	Mandible	Low
	64	M	Maxilla	Intermediate
	43	M	Mandible	Intermediate
	66	M	Mandible	Intermediate
	28	F	Maxilla	Intermediate
	66	M	Maxilla	Intermediate
	35	M	Maxilla	Low
	60	F	Mandible	Intermediate
Nagasaki et al. [15]	66	M	Mandible	Low
Reddy et al. [16]	66	F	Mandible	Low
	75	F	Mandible	Low
Maruyama et al. [17]	67	M	Mandible	NP
Wang et al. [18]	28	M	Maxilla	Low
	30	M	Maxilla	Intermediate
	23	F	Maxilla	Low
	30	F	Maxilla	Low
	30	F	Mandible	Low
	57	F	Mandible	Low
	30	F	Mandible	Low
This case	75	F	Mandible	Low

NP: not provided.

## Data Availability

Data supporting this research article are available from the corresponding author or first author on reasonable request.
